# Healthcare leaders’ use of innovative solutions to ensure resilience in healthcare during the Covid-19 pandemic: a qualitative study in Norwegian nursing homes and home care services

**DOI:** 10.1186/s12913-021-06923-1

**Published:** 2021-08-27

**Authors:** Hilda Bø Lyng, Eline Ree, Torunn Wibe, Siri Wiig

**Affiliations:** 1 SHARE - Centre for Resilience in Healthcare, Faculty of Health Sciences, University of Stavanger, N-4036 Stavanger, Norway; 2 Centre for Development of Institutional and Home Care Services in Oslo, PO box 4716, N-0506 Oslo, Norway

**Keywords:** Crisis driven innovation, Resilience in healthcare, Nursing homes, Home care services, Leadership

## Abstract

**Background:**

The Covid-19 pandemic introduced a global crisis for the healthcare systems. Research has paid particular attention to hospitals and intensive care units. However, nursing homes and home care services in charge of a highly vulnerable group of patients have also been forced to adapt and transform to ensure the safety of patients and staff; yet they have not received enough research attention. This paper aims to explore how leaders in nursing homes and home care services used innovative solutions to handle the Covid-19 pandemic to ensure resilient performance during times of disruption and major challenges.

**Methods:**

A qualitative exploratory case study was used to understand the research question. The selected case was a large city municipality in Norway. This specific municipality was heavily affected by the Covid-19 pandemic; therefore, information from this municipality allowed us to gather rich information. Data were collected from documents, semi-structured interviews, and a survey. At the first interview phase, informants included 13 leaders, Head of nursing home (1 participant), Head of Sec. (4 participants), Quality manager (4 participants), Head of nursing home ward (3 participants), and a Professional development nurse (1 participant), at 13 different nursing homes and home care services.

At the second phase, an online survey was distributed at 16 different nursing homes and home care services to expand our understanding of the phenomenon from other leaders within the case municipality. Twenty-two leaders responded to the survey. The full dataset was analysed in accordance with inductive thematic analysis methodology.

**Results:**

The empirical results from the analysis provide a new understanding of how nursing homes and home care leaders used innovative solutions to maintain appropriate care for infected and non-infected patients at their sites. The results showed that innovative solutions could be separated into technology for communication and remote care, practice innovations, service innovations, and physical innovations.

**Conclusion:**

This study offers a new understanding of the influence of crisis-driven innovation for resilience in healthcare during the Covid-19 pandemic. Nursing home and home care leaders implemented several innovative solutions to ensure resilient performance during the first 6–9 months of the pandemic. In terms of resilience, different innovative solutions can be divided based on their influence into situational, structural, and systemic resilience. A framework for bridging innovative solutions and their influence on resilience in healthcare is outlined in the paper.

## Introduction

A pandemic has a high probability of resulting in major consequences [1]. Covid-19, which was declared a pandemic by the World Health Organization (WHO) and hit Norway in March 2020, challenged the Norwegian healthcare system [2]. The Norwegian healthcare system, and healthcare systems worldwide, had to use their available staff, knowledge, and equipment resources to provide the best possible care for patients suffering from a new and unfamiliar disease, while preventing the virus from spreading among staff and patients. Most research on the Covid-19 outbreak has been directed towards hospitals and especially intensive care units [3].

However, nursing homes and home care services in charge of highly vulnerable patients have also been forced to adapt and transform to ensure the safety of patients and staff; yet, they have not received enough research attention. This paper aims to fill this research gap by investigating how leaders in nursing homes and home care services responded to the pandemic situation from a resilience and innovation perspective.

### Resilience and innovation in crisis

The ability of healthcare organizations to continue operations during a crisis relies on the organization’s resilience [4]. Developing resilience in healthcare would decrease organizations’ vulnerability to crisis, as they would be better prepared and more efficient, resulting in reduced disruption of the delivery of healthcare services [5]. Resilience in healthcare may be framed as the capacity to adapt to challenges, changes, and risks at different system levels to maintain high-quality care [6]. Based on this understanding of resilience in healthcare, the organization’s adaptive capacity is a vital source for ensuring resilience. The Covid-19 pandemic introduced a need for adapting, transforming, and reorganizing existing guidelines and practices to meet novel and upcoming requirements [2]. Creativity and innovative solutions were needed to develop new procedures, processes, work practices, and products. This is also supported by literature that describes innovation as a fundamental pillar of adaptive capacity, linking innovation and adaptive capacity to resilience [7, 8].

A pandemic introduces huge challenges and opportunities for both resilience and innovation in healthcare. Adaptations to ensure resilience in healthcare can take many forms, and the timeframe can be from seconds and minutes to long-term reorganizations that unfolds over years and decades [9, 10]. Macrae and Wiig [11] describe resilience in terms of time, space, organizations, and levels, providing a valuable framework for understanding resilience in time-sensitive situations, like a pandemic. *Situational resilience* refers to combining and activating existing sociotechnical resources to adapt to disruptive events [10, 11]. Situational resilience evolves from situated practices over seconds to weeks and relates to front-line situations where rapid mobilizing and problem-solving are needed. *Structural resilience* refers to the deliberate reorganizing, redesigning, and restructuring of sociotechnical resources based on monitoring organizational activities [10, 11]. The development of structural resilience is more time-consuming, extending to weeks and even years, and affects a larger group of people. The third and last type of resilience in this typology is the systemic type of resilience. It unfolds over months to decades and affects and disrupts the overall system. *Systemic resilience* is often formed based on macro-level initiatives and provides an overall reconfiguration of how sociotechnical resources are designed, produced, and implemented [10–12].

Barasa et al. [5] in their literature review of organizational resilience, proposed that resilience consists of 10 influencing factors: material resources, preparedness, information sharing, collateral pathways and redundancy, governance processes, leadership practices, organizational culture, human capital, social networks, and collaboration. Based on this understanding, the authors divided resilience into two forms: *planned resilience* and *adaptive resilience*. Planned resilience refers to planning and preparing for future crises, while adaptive resilience refers to responding to chronic stresses (everyday resilience) and acute shocks (crisis). As such, planned resilience represents proactive activities, and adaptive resilience reflects reactive responses.

*Innovation* can also be divided into different types based on their impact on the system. Some innovations are incremental solutions for improving situational practices and products, while others contribute to disrupting the entire industry. A crisis is traditionally described as an unexpected event with accompanying uncertainties, posing a threat to humans and places, where decisions need to be made at a fast pace [3]. This leaves few opportunities for careful and resource-intensive development of new solutions, a feature known to be important in the early phases of innovation development [13]. To deal with the crisis, there is a need for novel and creative ideas to solve upcoming challenges in an innovating manner, as the established ways of working are no longer appropriate for handling the situation. The literature on crisis-driven innovation has focused on this issue by seeking to understand how people develop innovations in crisis [13–15].

*Crisis-driven innovation* describes some new characteristics of the innovation process. The extreme conditions associated with a crisis, such as a pandemic, enforce a need to rethink traditional approaches. The innovation process shifts to a laboratory approach where new solutions are explored within and across the organizational context [13]. The laboratory way of innovating relies on rapid prototyping, learning, diffusion, and, therefore, more experimentation compared to traditional innovation processes [13]. Of particular importance is the ability to innovate through re-combining existing knowledge [16–18]. As knowledge of the crisis is yet to be developed at the outbreak stages, combining existing knowledge/solutions from other organizations and sectors with situated knowledge/solutions provides a resource for innovation. As such, innovation can be developed without the need for extensive testing and prototyping.

However, innovative solutions are not a magic pill to overcome and manage well in a crisis. Leadership is needed to introduce and implement new procedures, practices, equipment, and safety tools in the organization and is a critical resource for success in terms of resilience [4]. This understanding is also reflected in the resilience factors proposed by Barasa et al. [5], where two factors are explicitly directed at leadership (governance processes and leadership practices), and the remaining eight factors are highly affected by the quality of the leadership. The national Norwegian Corona Evaluation Panel identified municipalities as key in the handling of the pandemic, despite limited resources and lack of preparedness (materials like Personal Protection Equipment (PPE) and staff) [2]. Examining how nursing homes and home care services, governed by Norwegian municipalities, managed to provide appropriate care at the outset of the Covid-19 pandemic would help us understand how these healthcare leaders introduced and implemented new innovative solutions into their organization to ensure resilience in healthcare.

### Aim and research question

This study was a part of the research project titled *Leader experiences of the Covid–19 pandemic in nursing homes and home care services*. This paper aimed to explore how nursing homes and home care leaders used innovative solutions to handle the Covid–19 pandemic to ensure resilient performance during times of disruption and major challenges to quality in service provision.

The following research question guided the research: What type of innovative solutions did nursing home and home care leaders use to ensure resilience in healthcare during the Covid-19 pandemic?

## Methods

### Research design and setting

Due to the lack of empirical data of leadership in nursing homes and home care services in terms of innovations during the Covid-19 pandemic, we chose a qualitative case study design [19, 20]. Our empirical case was one large city municipality in Norway. The municipality of choice was one of the Norwegian municipalities most heavily affected by the Covid pandemic; therefore, it was expected to provide rich information to understand the phenomenon under study, in accordance with theoretical sampling guidelines [20]. The municipality was recruited in collaboration with a Centre for Development of Institutional and Home Care Services located in the region of the municipality. A co-researcher TW contributed to contact leaders and obtained their consent to participate in the study.

Nursing homes and home care services are the responsibility of the municipalities in Norway. Furthermore, most nursing homes and home care services in Norway are public and non-profit institutions (14 public and 2 private organisations within this dataset). By law, Norwegian municipalities must organize their healthcare services to ensure sound professional practice. The municipalities and their leaders are responsible for complying with the Regulation on Management and Quality Improvement in the Healthcare Services and for establishing safety management systems [21–23].

Nursing homes and home care services are separate institutions, although both are subject to the municipality. The healthcare system in Norway is set up for elderly and chronically sick patients to live at home for as long as possible. This means that home care services perform a range of care, from minor supervision to quite advanced nursing. Correspondingly, residents at nursing homes are elderly or chronically sick patients that are found too sick or disabled, temporarily or permanently, to stay at home. Meaning that nursing homes perform advanced care for their patients.

### Data collection and sample

We recruited leaders from nursing homes and home care services in the case municipality. The recruitment was supported and organized by the co-researcher (TW), who works at the Centre for Development of Institutional and Home Care Services within the case municipality. Having a co-researcher with contextual understanding was found highly valuable for legitimacy in the recruitment phase. All nursing homes (totalled 46) and home care services (15 districts) of the chosen case municipality were invited to participate in the study [24]. The different nursing homes and home care services themselves signed up for participation. The empirical data was collected in a two-phase process using triangulation methods of interviews with 13 nursing home and home care leaders and an online survey administered to 16 institutions (remaining leaders at the 13 interview institutions and 3 additional institutions). Sample size was based on information power guidelines by Malterud et al. [25]. All informants were leaders before the outset of the pandemic and found to be highly informative sources for this study.

At the first phase, interviews were conducted from September to November 2020. All interviews were semi-structured, using broad questions from an interview guide asking the participants to discuss topics on *leadership, practices, resources, innovation, and knowledge sharing*. The following questions from the overall interview guide (data corpus) were included in the data set for this study:

1. *Demographic characteristics (age, education, experience, position)*
2. *How was the Covid-19 pandemic handled at your nursing home/home care service?*
3. *What factors do you think were successful in handling the pandemic at your institution?*
4. *What types of innovative solutions were developed/implemented in response to the pandemic at your institution, and who came up with the innovative solution/idea?*

All interviews were conducted online by videophone (Teams), as the pandemic was ongoing and traveling and visiting at the informant’s workplaces could not occur. The interviews lasted from 30 to 60 min.

At the second phase, a short survey covering the same topics as the interviews, mainly consisting of open-ended questions and some close-ended Likert-type questions (1–5), was sent to leaders holding other leadership positions at the participating study sites. We found innovative solutions, during the interview phase, to address highly local situations. The inclusion of other wards, departments, and sections in the survey, therefore allowed for new information of innovative solutions and hence broadened the perspectives and nuances of the data. The survey yielded 22 individual responses. This article focused on responses from the following survey questions:

*Were new innovative solutions developed as responses to the pandemic?*

*If yes, then*:

1. *Give a brief description of the innovative solutions*
2. *Who came up with the idea and what was the origin of the idea?*
3. *How was the innovative solution implemented?*

All participants signed written informed consent. In the survey, the first question asked the participants to provide their consent to participate in the study.

### Participants

The interviewed participants included 13 leaders. Eight participants held leadership positions in nursing homes, and five participants were leaders in home care services. The age of the interviewees ranged from 36 to 55, with a mean of 44.17. Types of leadership positions in the sample included Head of the nursing home (1), Head of Sec. (4), Quality manager (4), Head of department (3), and a Professional development nurse (1).

Participants responding to the survey consisted of healthcare leaders (84 % nursing homes vs. 16 % home care services) aged 40–49 (57 %), 50–59 (38 %), and 30–39 (5 %). Types of leadership positions included Quality manager (29 %), Head of nursing home/department (43 %), and Head of nursing home ward/Sec. (29 %). Years of leadership experience were classified as less than one year (5 %), 1–5 years (24 %), 6–10 years (19 %), 11–15 years (24 %), 16–20 years (14 %), and 21 years or more (14 %).

According to the Norwegian context, the above-mentioned leadership positions refer to the following responsibilities: Head of institution is the overall manager of the nursing home, and holds the full responsibility for staff, competence and economy. The Head of ward refers to the leader of a ward at nursing homes, where they possess responsibility for staff, competence, and economy in accordance with authorizations from the Head of institution. Head of department in home care services includes the responsibility for several sections that are subject to the district. Correspondingly, Head of section is a leader of a smaller district in home care services who reports to the Head of department. The Quality manager oversees that the nursing home or home care service is providing high quality services and furthermore complies to laws, regulations, and guidelines. The professional development nurse has the responsibility for competence development at the department/ward/section. The included leadership positions represent the traditional organization for nursing homes and home care services, and thus a diversity of leadership roles.

### Data analysis

All interviews, 13 in total, were audio-recorded and transcribed verbatim. The transcribed interviews were later combined with qualitative open-ended survey questions to make up the full dataset for this study. As data collection was finalized, data analysis started in accordance to inductive thematic analysis [26]. The term innovation in healthcare in the interview guide and within the survey was defined as *“innovations need to be perceived as new by a portion of key stakeholders”* [27]. However, this definition does not specify the type and form of innovations and, as such, allowed for an inductive analysis of various types of innovation.

Based on the thematic analysis framework by Braun and Clarke [26], the following analytical steps were conducted. First, all data in the dataset were transcribed and read by all researchers. Second, the first author generated initial codes for various types of innovations found in the material. The authors discussed the results from this first round of coding during the analysis meetings. In the third phase, the initial codes were converted into themes (technology, practices, services, and physical) reviewed in the fourth phase during the analysis meeting with all researchers, resulting in Table 1. The fifth phase included a revision of themes and an analysis of how the different types of innovative solutions contributed to resilience in healthcare. The first author developed and proposed Table 2 to all authors, achieving a consensus. Differences in opinion among the involved researchers were solved by reviewing the original data, which were found to develop consensus for all differences experienced in this study.

**Table 1:** Different types of innovation used by leaders in primary care

Themes	Codes
Innovative technological solutions for communication	❖ Workplace by Facebook ❖ Teams ❖ Patient administration software ❖ Staff communication software ❖ Communication unit for interaction between next-of-kin and patients (video, messages, and pictures) ❖ Recruitment of new staff through Facebook and Instagram ❖ Software for infection tracing developed by the municipality
Innovative technological solutions for remote care	❖ Medication dispensers ❖ Weight control ❖ Digital consultancy
Innovative solutions for practices	❖ Procedure and Protection Equipment controllers ❖ The establishment of a response team ❖ Leaders acting as knowledge brokers ❖ Inhouse testing of Covid-19 ❖ Checklists where healthcare professionals (HCPs) had to confirm receiving Covid information ❖ External actors assuring the quality of changes ❖ Different entrances, where HCPs got a message the previous day explaining which entrance to use the next morning ❖ Visitor assistant ❖ Recruitment of laid-off workers (from hotels and restaurants)
Innovative solutions for services	❖ Outside area for hosting concerts and shows. ❖ Use of laid-off workers from service industries (aviation, hotels, restaurants) to serve food and communicate with the patients.
Innovative solutions for the physical environment	❖ Changing zones for healthcare professionals to dress up in protective equipment ❖ Areas for teaching (outdoors) ❖ Proving different entrances ❖ Dividing into infection and non-infection wards ❖ Motion detectors for monitoring patients with dementia

**Table 2:** Type of innovation for different types of resilience

Moments of resilience	Type of innovation
**Situated resilience**	**Recruitment through social media** **Recruitment of resources from service industries** **Using different entrances in/out of the building** **Teams, Workplace** **Outdoor teaching areas**
**Structural resilience**	**Remote care** **Software communication platform** **Procedure and Protection Equipment Controllers** **Inhouse testing of Covid19** **Visitor assistants** **Outside concerts** **Establishing infection and non-infection wards** **Changing zones for protection equipment** **Infection equipment trolleys** **Motion detectors**
**Systemic resilience**	**Infection tracing software** **Response team**

## Results

The analytical process results are illustrated in Table 1, summarizing innovation themes and the codes forming the themes. The results display a high degree of adaptive capacity and a myriad of new solutions being developed and implemented to adapt to the major disruption caused by the pandemic. Leaders elaborated on adopting new technology, establishing new work practices, reorganizing service provisions, and adapting to the physical environment to practice effective infection control.

### Innovative solutions for communication

Most of the communication technology did not concern the introduction of new technology but rather the adoption of existing technology to new contexts. Although software like Teams, for example, is familiar, this tool was not used in work situations in nursing homes and home care services. Teams were mostly introduced, during the pandemic, to facilitate communication between actors at the meso level (leader meetings) and between the macro and meso level (e.g., dissemination of new information and guidelines) but were not considered appropriate for discussing sensitive information about patients.

Leaders distributed the continuous flow of information from national and regional authorities to the front-line staff through various channels: email, SMS, printed forms in staff rooms, and the Workplace platform. Workplace by Facebook was considered a valuable platform for distributing new knowledge and information to staff due to its availability (app on their mobile phones) and ability to monitor whether the information was read and by whom.

*“And then eventually, we have started using a software called Workplace, where we post information. We make “Corona-news» every week, which I’m responsible for. I then gather information from the municipality, the Norwegian Institute of Public Health, the municipal nursing home service, about new and updated procedures and then post it” (nursing home).*

The Covid-19 pandemic also challenged the staffing situation, where up to half of the staff had to stay at home due to infection or quarantine. This challenge led the leaders to seek new ways of recruiting personnel. Instead of calling employment agencies, which was the first choice of all nursing homes, Instagram and Facebook were used to announce their need for new staff. Using a different channel to seek new people allowed new groups of people, like laid-off workers from other Covid-19 affected industry sectors, such as hotels, cabin crews, restaurants, to apply. These laid-off workers were then temporarily hired to help out with non-medical work tasks.

Unlike the technology mentioned above, a new health-technology unit was introduced to facilitate interaction between patients and next-of-kin at nursing homes. This communication platform, an innovation by a private med-tech company, was specially developed for elderly patients with cognitive and/or physical disabilities. The nursing home unit was designed as an analog tv-screen, with only a single power switch and easy functionality (no username and password) for elderly patients to operate. The app designed for the next-of-kin on mobile phones required username, password, and administration rights. Next-of-kin could initiate video communication with the elderly and post pictures for the elderly to look at when alone. During the pandemic, the nursing homes at times had to close for visitors, based on national healthcare regulations. As a response to these regulations, some nursing home leaders signed up to be test locations for this technology, thereby providing a way for the next-of-kin to keep in touch with their loved ones at the nursing home.

The pandemic also led to new technology development. Particularly infection tracing was perceived as a time - and resource-intensive challenge for the leaders. Therefore, software for infection tracing was developed by the municipality to ease this process. The development was initiated based on feedback from leaders and front-line workers, and the software allowed for an integration of information about all work shifts at different home care services and nursing homes, patient journals, and the population registry. As such, close contacts of infected individuals could be traced and contacted more efficiently.

*“We are using XX [infection tracing system], which was developed during the pandemic….It has eased the work a lot. And because the entire municipality is using the same system, we can easily transfer patients between us….Everything is documented here. All is included”* (homecare service).

### Innovative technology solutions for remote care

Not all technological solutions were related to communication. Other technological solutions involved the remote care of patients to limit the number of home visits in the home care services. Like communication technology, most remote care technologies were available on the market before the pandemic, although it was not necessarily adopted in home care services. The traditional way of monitoring patient medication is to observe that patients took their medication during daily visits from home care services. Electronic medication dispensers allowed to monitor medication compliance remotely. If the patient did not take his/her medication, the home care service was notified, and they would call the patient to make him/her aware of this situation. Correspondingly, patient’s weight could also be monitored by implementing welfare technology.

*We have become much more aware of remote care monitoring in general, like using electronic medication dispensers. Controlling weight could also be a way of using remote care. We are given a tool that allows us to do things differently”* (home care service).

### Innovative solutions for practices

Healthcare leaders described a marked change in their attitude towards healthcare practices during the Covid-19 pandemic, as they had to shift their focus from treating infected patients to protecting non-infected patients. Some leaders introduced in-house Covid-19 testing of staff and patients and developed their own protocol for test practices.

Another example of new practices focused on protecting non-infected patients from the virus involved creating a dedicated role to ensure healthcare professionals wore protective equipment according to procedures. This control person acted as a *Procedure and Protection Equipment Controller* in the changing areas. Nursing home leaders soon discovered that just providing their staff with appropriate protection equipment was not enough to prevent the virus from spreading, as they frequently witnessed its improper use. Due to the lack of staff at certain times, the leaders altered their daily work schedules to be present at the sharp end and make sure their staff wore their protection equipment appropriately.

*“We had a present leadership. When they arrived at their shift in the morning, we met with them, talked to them, and took them up to the infection wards. We also met them when they came back from the infection wards and talked to them again”* (nursing home).

Additionally, the leaders were responsible for providing and distributing protection equipment to their staff, which was experienced as a challenge at the outset of the pandemic due to the lack of protective equipment worldwide. The leaders had to keep the stock of protection equipment locked, as staff, due to fear, tried to supply themselves with more than what were assigned for their work tasks.

Another area in need of new practices was next-of-kin interaction. Nursing homes, for some periods, had to close to visitors, making digital communication between patients and next-of-kin the only option. However, when the infection rate within the municipality was lower, next-of-kin were allowed to meet their non-infected relatives under strict regulations. To ensure compliance with regulations, a position as visitor assistant was established, as described in the quote below.

*“First, they (next-of-kin) book a specific appointment if they want to visit. And then we have a person who talks to them (on the phone) and makes a list of appointments. When they arrive, they are met by this visitor assistant, who uses the checklist to clarify whether they have been in contact with someone (infected) or whether they have been traveling, and all this. Then they direct them (next-of-kin) to the ward and inform them about all our procedures. In the evenings, we have some students to help us with this new routine” (nursing home).*

Nearly all included institutions in this study created some form of response team to react to the pandemic. In home care services, the response team was responsible for providing care in the homes of infected patients. As such, the number of staff in contact with infected patients was limited. In nursing homes, specific response teams were assigned to handle infection at the ward, as the following quote illustrates:

*“I think the most important thing (for succeeding) is to have a response team that knows the tasks that need to be done. One person prepares the equipment, one is dividing the ward into infection and non-infection zones, and one is tracing the infection. I think it is important to place the responsibility for the specific tasks within the team”* (nursing home).

### Service innovations

Healthcare professionals soon realized the lack of resources available to keep up with patient-centred care during the pandemic. Some leaders therefore decided to hire people with no healthcare experience but with strong people skills. As the pandemic affected the service industries hard, many employees in, e.g., hotel, aviation, and restaurant industries, were laid-off and provided valuable resources for the nursing homes, as described in the following quote.

*“So, we had to think logically; what do we need at the wards where we lack nurses and healthcare workers? We need someone who can talk to the patients, who can serve food, make breakfast, serve dinner….And one who is used to working in the hotel industry, who works with people, who is kind, is pleasant, service-minded, right? We needed this kind of people. And it turned out to work well. So, they (people from service industries) got training at the non-infected wards”* (nursing home).

Another area heavily affected by the reprioritization of resources, due to the pandemic, concerned social activities offered to patients. Therefore, some leaders provided their patients with innovative solutions for outdoor concerts and shows (like ballet), where the patients could watch from their balconies, as described in the following quote.

*“But we arranged some outdoor concerts during the spring, so we managed to have some activity all along, but then mostly outdoors, due to the guidelines. We were even so lucky that we got the opera to visit us, and the opera-ballet group danced outside. We put up a stage.”* (nursing home).

### Innovative solutions in the physical environment

In nursing homes, the physical environment had to undergo huge changes, like establishing infection and non-infection wards, protective equipment changing areas, areas for disposing infection materials, different entrances for staff at infection and non-infection wards, and areas for teaching and practicing infection procedures.

Providing safety for patients with cognitive impairment was especially challenging, as these patients were often difficult to isolate in infection/non-infection wards. This challenge resulted in an innovative solution. A nursing home leader started to use motion detectors to monitor whether the cognitively impaired patients crossed between infection and non-infection wards.

*“So, we used a lot of motion detectors. If patients crossed the line, we would immediately get a signal. It is on the phone (the signal), and then we know straight away that there is a risk of somebody leaving the ward, right?”* (nursing home).

Another physical innovation at the wards was the development of portable infection equipment trolleys to make the treatment of infected patients more efficient. These infection equipment trolleys included all the necessary equipment to care for infected patients, like gloves, bin bags, facemasks, tissues, disinfection, glasses, saturation meters, thermometers, and posters illustrating safe use of the equipment and the current procedures. The infection equipment trolleys improved in functionality over time during the pandemic, as the following quote demonstrates.

*“We did not have these trolleys before the pandemic, so in the beginning, we had to improvise by using bedside tables and everything else that could be useful (laughing).” (nursing home).*

## Discussion

This study explored innovative solutions that leaders in nursing homes and home care services adopted to handle challenges introduced by the Covid-19 pandemic. Our results align with the national Corona Evaluation Panel’s findings, which reported that the Norwegian municipalities had effectively handled the pandemic crisis despite the lack of preparedness and protective equipment in the early phase of the pandemic [2]. The results section described the role and nature of the innovative solutions. This section focuses on the impact of these innovative solutions for different types of resilience. The link between the type of innovation and resilience is depicted in Table 2, informed by the resilience framework by Macrae and Wiig [11] of situated, structural, and systemic resilience.

As a strategy to disruptive events, *situated resilience* uses existing resources and takes place within seconds or weeks [9–11]. Innovative solutions seeking to enhance situated resilience included the adoption of existing technology to deal with the emergency. A challenge in the early phases of the outbreak was the lack of staff due to quarantine and infection. Therefore, nursing home and home care leaders quickly had to find ways to obtain new resources. They used social media, like Facebook and Instagram, to get in touch with possible new resources, providing situated resilience for the institution. Following Barasa et al. [5], this can be described as adaptive resilience through collateral pathways, referring to alternative routes of actions to ensure resilience.

However, the new practice of hiring personnel through social media was not perceived as a permanent practice adopted within the organization over time but rather as a temporary practice innovation that provided resilience in a specific situation. The new practice of using existing technologies, like Facebook and Instagram in the example above, could be initiated in minutes, and the solution was installed rapidly at the front-line [10, 11].

To prevent the virus from spreading among healthcare professionals, the use of existing communication technology, like Teams, allowed for shared communication and information transfer. Furthermore, collaborative learning on the appropriate use of protective equipment by healthcare professionals had to be located outdoors and offered in small groups to comply with guidelines and procedures. All these actions were initiated quickly, utilizing the existing resources in response to disruptive events requiring quick solutions. Furthermore, these innovative solutions complied with the responding potential described by Hollnagel [28] and adaptive resilience by Barasa et al. [5], functioning as a tactic for narrowing the situated demand-capacity misalignment [9].

*Structural resilience* refers to the restructuring and reorganizing of resources and practices [10, 11, 29]. These tactics are not as quick to implement and rely on more effortful development. Some innovative solutions in this research were found to provide structural resilience.

First, monitoring [28] patient and staff safety strategies, like installing remote care technology in home care services, utilizing specific visitor assistants and protective equipment controllers, and implementing in-house testing of Covid-19, were all based on self-organizing procedures found to provide structural resilience.

Second, some structural resilience strategies heightened anticipation [28], like developing an infection equipment trolley. Furthermore, establishing infection and non-infection wards and using motion detectors to prevent patients with cognitive impairments from entering/leaving infection wards are examples of innovative solutions for structural resilience. These tactics were proactive actions to prevent the further spread of the virus, thereby strengthening resilience factors, like preparedness and awareness, by reorganizing resources, which Barasa et al. [5] defined as planned resilience.

Third, structural resilience also included response tactics [28], like the facilitation of outdoor concerts and shows (responding to a need for activization of patients). Unlike situational resilience responses, these tactics were formed based on a reorganization process, as the outdoor concerts required planning and reorganization of practices at the nursing home. As such, these tactics represented collateral pathways for adaptive capacity by using alternative routes of action in response to the pandemic [5].

*Systemic resilience* refers to tactics developed by the reformulation of design, production, and dissemination of resources in sociotechnical systems to influence the overall system [9–11]. Systemic resilience relies on a thorough development evolving over months to decades. Some innovative solutions found in this study, like new infection tracing software developed by the municipality, produced an overall effect for all institutions. The infection tracing software was a government process for initiating preparedness and a source of planned resilience [5]. Furthermore, all organizations created a response team to be better prepared for new Covid-19 outbreaks and future pandemics. A present response team provided efficient management of infection outbreaks among staff and patients, as all team members had clear roles and responsibilities. As such, the response team and the infection tracing software are solutions that will remain across institutions over time.

The focus of the crisis-driven innovation literature has mostly been directed towards output (e.g., product, services, and practices) and innovation processes in crisis [13, 14]. This view was also reflected in this study, where different innovative solutions are described in detail. However, to the best of our knowledge, the effect of crisis-driven innovation for resilience in healthcare has not been described in the literature, even though innovation has been highlighted as a facilitator of resilience [7]. This paper contributes to this literature by providing an empirical understanding of the effect of crisis-driven innovation on resilience in healthcare.

Limitations.

This study has some limitations that should be acknowledged. First, the sample size was relatively small. However, this was compensated by collecting data through both interviews and a survey. We consider the information collected in the study as sufficient, as the group of managers provided rich information highly relevant to our research question [25]. Second, data collection was conducted 6–9 months after the first lockdown in Norway. This could influence the results, as the leaders had overcome some parts of the initial disruptions. Third, this was a single case study of one municipality only. However, this was one of the most affected municipalities in Norway. Further studies could focus on investigating leaders from different locations, considering different infection grades, sizes, and success in handling the pandemic. As the pandemic remains a challenge worldwide, new studies should be performed to explore innovative solutions at later stages of innovation development.

## Conclusions

 This paper explored how leaders in nursing homes and home care services used innovative solutions to handle the Covid–19 pandemic in a Norwegian municipality through a combined resilience and innovation perspective. Nursing home and home care leaders implemented several innovative solutions to ensure resilient performance. Innovations in response to the Covid-19 pandemic can be grouped into the following themes: *Technology*, *Practices*, *Services*, and *Physical innovations*. In terms of resilience, these different innovative solutions can furthermore be grouped, based on their influence, into:

1. Innovative solutions for *situational resilience*, where existing resources were used in new situational contexts (e.g., social media, outdoor teaching places, recruitment from service industries). As these solutions relied on existing resources, they could be rapidly integrated within the institution in emergencies.
2. Innovative solutions providing *structural resilience*. Their development were more time-consuming, and they could be used to monitor, respond, and anticipate, all known as valuable resilience potentials.
3. The innovative solution resulting in *systemic resilience* transformed services across the entire industry; therefore, they will continue to be used after the pandemic.

This study focused on nursing home and home care service leaders’ perspectives during the first period (within 6–9 months) of the Covid-19 outset. The pandemic outset required innovative solutions and a formidable adaptive capacity of healthcare professionals. As such, this study contributes to our understanding of how leaders in nursing homes and home care services used innovative solutions to provide sound healthcare to their patients. Furthermore, this paper builds a conceptual bridge between crisis-driven innovation and resilience in healthcare, illustrating that both theoretical disciplines are of importance in a crisis like the Covid-19 pandemic.

## Data Availability

The datasets used and analysed in the current study are available from the corresponding author upon request.

## Abbreviations

HCP: Healthcare professionals
WHO: World health organization
NSD: Norwegian Centre for Research Data
